# What is the link between the antidepressants, the transcranial magnetic stimulation and the peripheral vascular endothelial growth factor?

**DOI:** 10.1192/j.eurpsy.2022.668

**Published:** 2022-09-01

**Authors:** J. Lazáry, M. Elemery, S. Kiss, L. Pogany, G. Faludi

**Affiliations:** 1 Nyírő Gyula National Institute of Psychiatry and Addictions, Department Of Psychiatry, Budapest, Hungary; 2 Semmelweis University, Janos Szentagothai Neuroscience Doctoral School, Budapest, Hungary; 3 National Institute of Mental Health, Neurology and Neurosurgery, Department Of Biological Psychiatry, Budapest, Hungary

**Keywords:** depression, rTMS, non-responders, treatment resistant depression, VEGF

## Abstract

**Introduction:**

Vascular endothelial growth factor (VEGF) has been implicated in mediating the effect of antidepressants (AD) and electroconvulsive therapy on depression since it plays a significant role in the neurogenesis. However, the serum VEGF level has not been investigated so far in association with rTMS treatment in patients with major depressive disorder (MDD).

**Objectives:**

The aim of our study was to compare the effect of the antidepressants and of the repetitive transcranial magnetic stimulation on the serum vascular endothelial growth factor and its association with the responsiveness to the treatments.

**Methods:**

A dataset of 50 patients with TRD who were treated with AD (n=33) and bilateral rTMS for 2x5 days (n=17) was analysed (sample ’rTMS&AD’). Montgomery-Asberg Depression Scale (MADRS) was used for monitoring the symptom changes. The serum VEGF levels and symptoms were assessed on the first (V1), on the 14th (V2) and on the 28th day (V3). The VEGF levels were measured by ELISA assay.

**Results:**

The baseline VEGF levels were significantly higher in non-responders both in the rTMS&AD (p=0.04) and AD samples (p=0.02) compared to responders. The MADRS reduction and the changes in VEGF levels between V1 and V3 were significantly associated in responders only in the AD&rTMS sample (p=0.03). The baseline VEGF level has been proven as a significant predictive factor of treatment response in the total sample (p=0.018).

**Conclusions:**

The baseline VEGF level can be a predictive factor to be a non-responder to different treatments. Change of the VEGF level is associated with the improvement of depressive symptoms only due to rTMS.

**Disclosure:**

This study was supported by the FK 131315 grant of the National Research, Development and Innovation Office, Hungary. Authors declare no conflict of interest.

